# Supplementary material for: Spatio-temporal dynamics enhance cellular diversity, neuronal function and further maturation of human cerebral organoids
Source: Commun Biol. 2023 Feb 14;6:173. doi: 10.1038/s42003-023-04547-1 (PMC9926461; doi:10.1038/s42003-023-04547-1)
Supplement: Supplementary file 2 — Supplementary Information [file 42003_2023_4547_MOESM2_ESM.pdf]

Supplementary Figures and Tables

Spatio-temporal dynamics enhance cellular diversity, neuronal function and further maturation of human cerebral organoids

Pelin Saglam-Metiner[1], Utku Devamoglu[1], Yagmur Filiz[1], Soheil Akbari[2], Goze Beceren[1], Bakiye Goker[3], Burcu Yaldiz[1], Sena Yanasik[1], Cigir Biray Avci[3,4], Esra Erdal[2,5], Ozlem Yesil-Celiktas[1,✉]

[1]Department of Bioengineering, Faculty of Engineering, Ege University, 35100, Izmir, Turkey

[2]Izmir Biomedicine and Genome Center (IBG), Dokuz Eylul University Health Campus, 35340, Izmir, Turkey

[3]Department of Medical Biology, Faculty of Medicine, Ege University, Bornova, Izmir, 35100, Turkey

[4]Department of Stem Cell, Institute of Health Science, Ege University, Bornova, Izmir, 35100, Turkey

[5]Department of Medical Biology and Genetics, Faculty of Medicine, Dokuz Eylul University, 35340, Izmir, Turkey

✉ ozlem.yesil.celiktas@ege.edu.tr

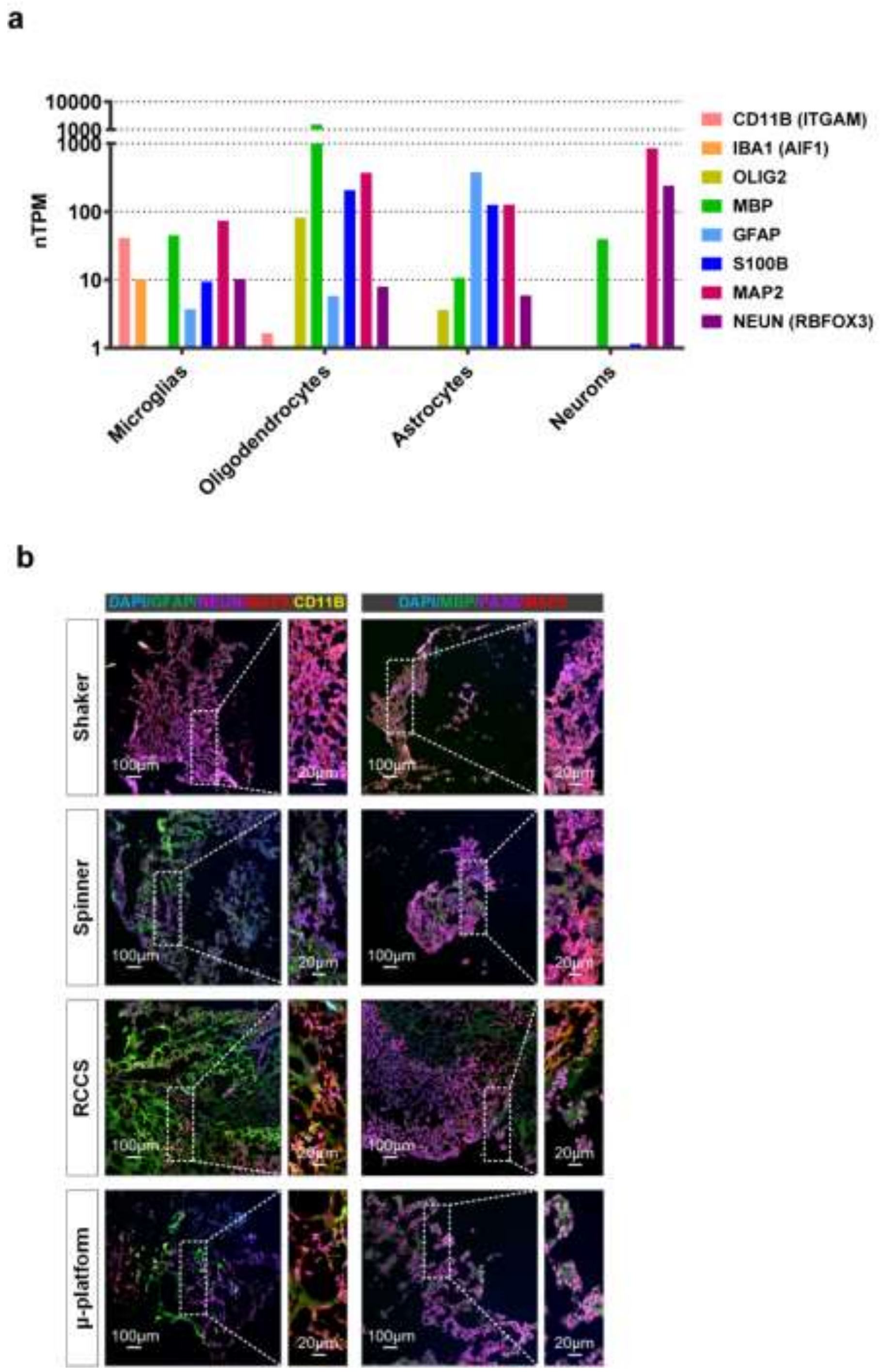

Supp. Fig. 1 a The normalized RNA expression levels of specific neuronal markers in different cell types, nTPM: normalized transcripts per million (*Human Protein Atlas V21.0 proteinatlas.org;* Karlsson et al., 2021) **b** Immunofluorescence staining of specific neuronal/glial cell markers (NEUN, MAP2, PAX6, GFAP, CD11B, MBP) on day 60 of organoids matured in dynamic systems (scale bars=100 µm for 10x and 20µm for 25x magnification images, independent replicates=3, Zeiss LSM 880).

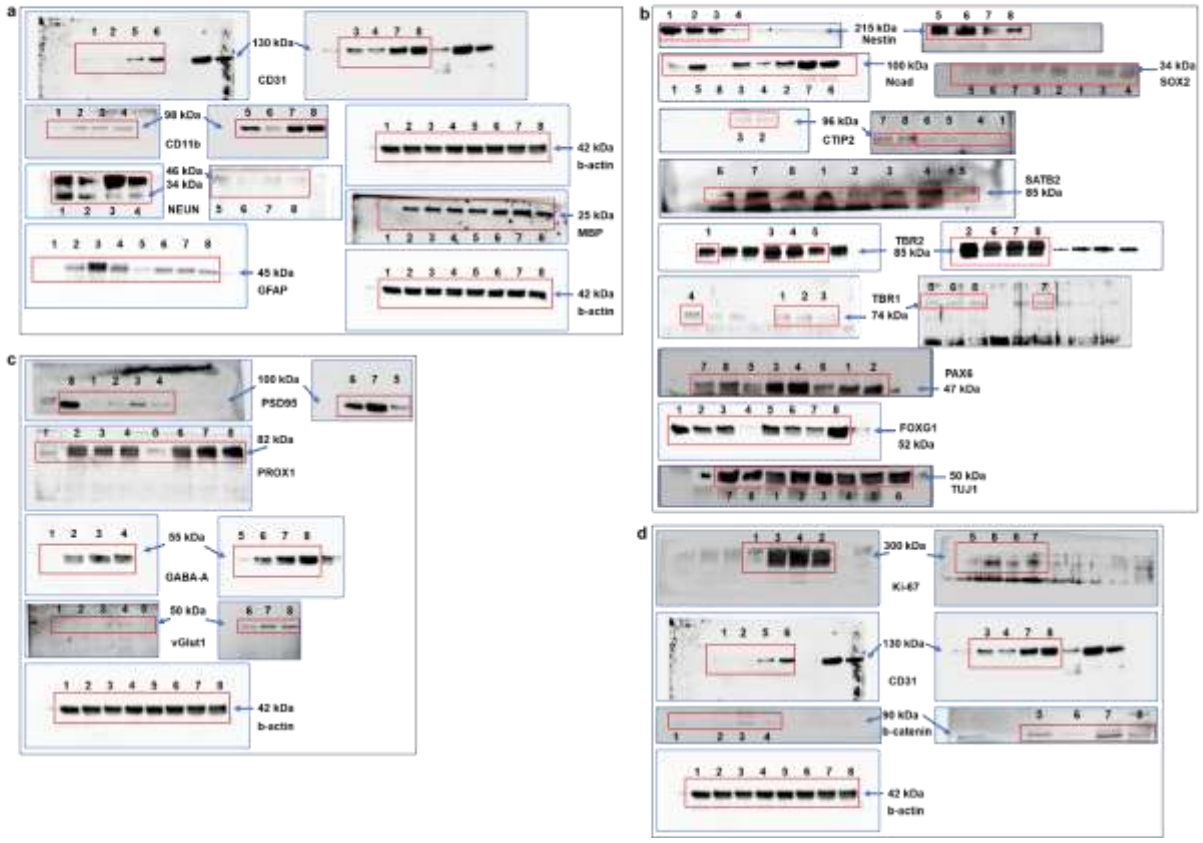

Supp. Fig. 2 Original western blot images. a Neuronal proteins, **b** specific maturation proteins, **c** advanced maturation proteins and **d** survival proteins of cerebral organoids matured in shaker (1,5), spinner (2,6), RCCS (3,7) and µ-platform (4,8) at day 60 and 120, respectively (independent replicates=2).

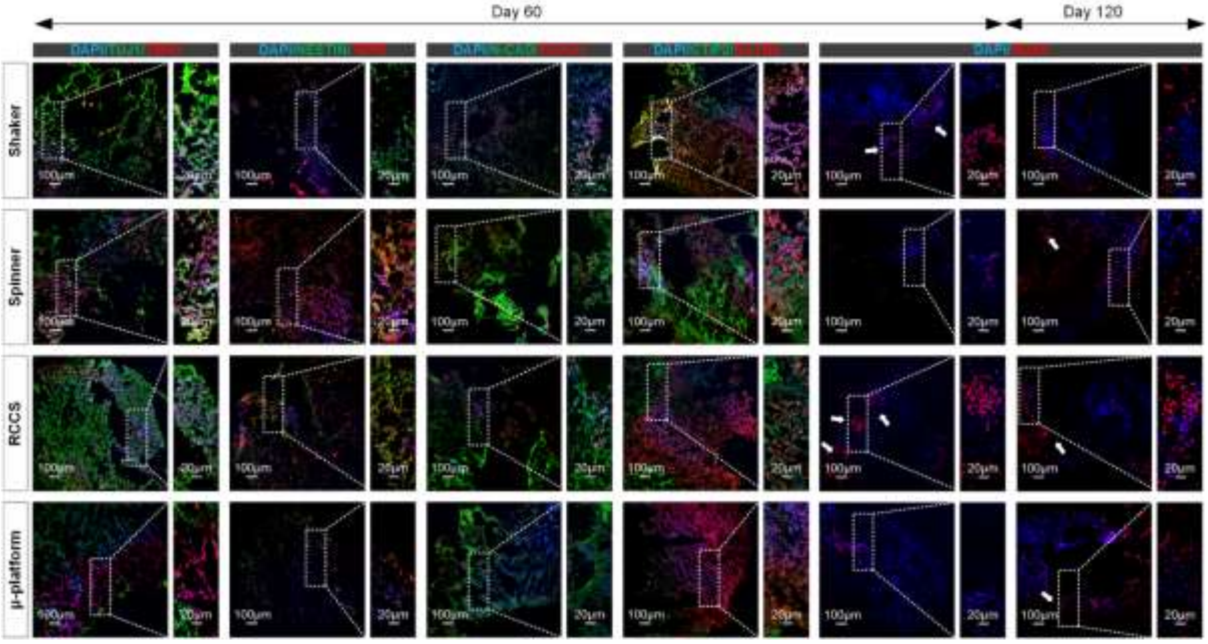

Supp. Fig. 3 Immunofluorescence staining of cerebral organoid maturation markers (TUJ1/TBR1, NESTIN/TBR2, N-CAD/FOXG1, CTIP2/SATB2 and SOX2) on days 60 and 120 of organoids matured in dynamic systems, white arrows indicate neural rosette-like structures (scale bars=100 µm for 10x and 20µm for 25x magnification images, independent replicates=3, Zeiss LSM 880).

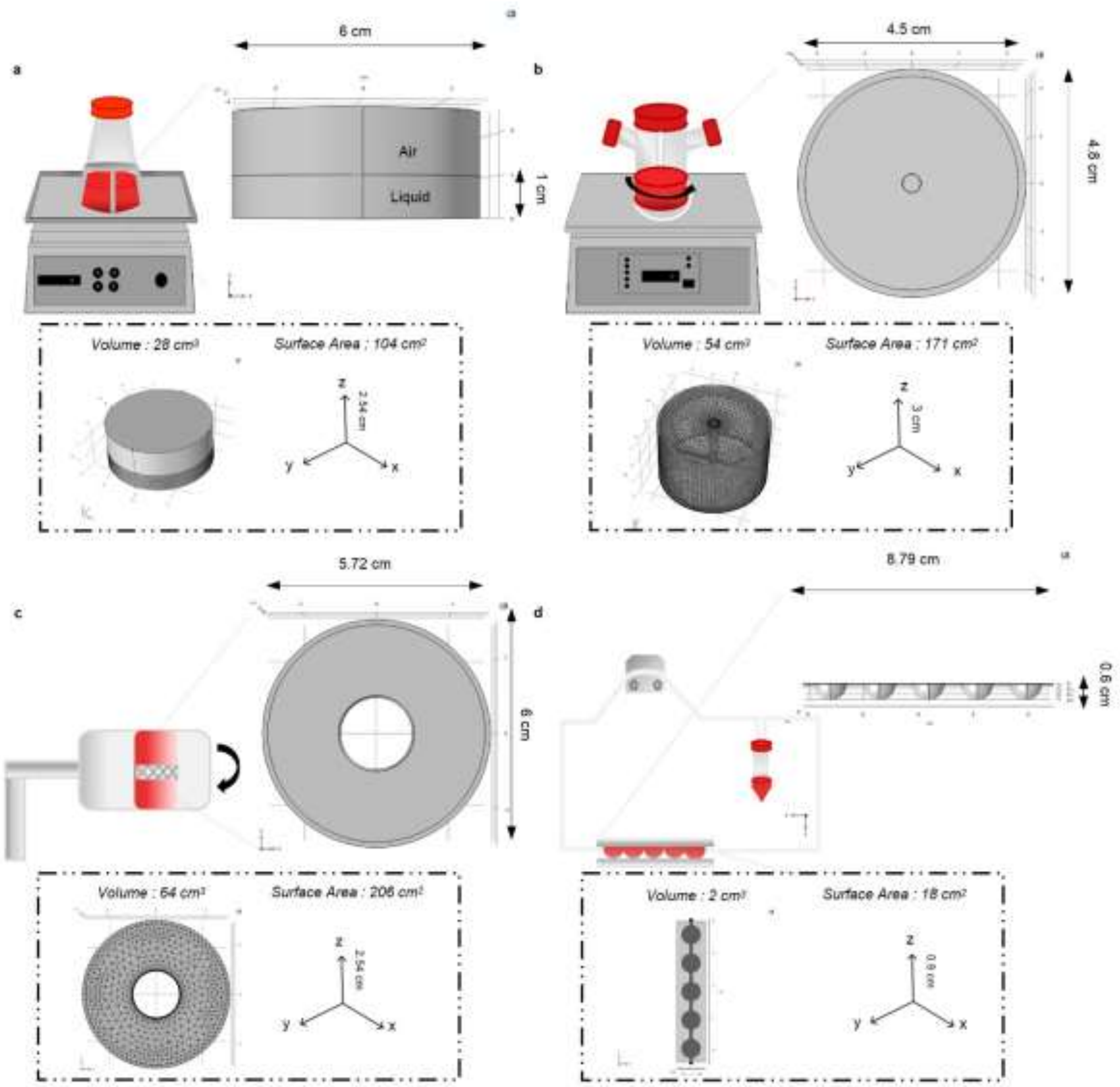

e

CFD Module/Physics type	Equations
Laminar Flow, Navier-Stokes Equation	$\rho(\mathbf{u}.\nabla)\mathbf{u} = \nabla[-p\mathbf{I} + \mu(\nabla u + (\nabla u)^T)] + \mathbf{F}$ $\rho\nabla.u = 0$
Turbulent Flow, k- ε	$\rho(\mathbf{u}.\nabla)\mathbf{u} = \nabla[-p\mathbf{I} + \mathbf{K}] + \mathbf{F} + \rho\mathbf{g}$ $\rho\nabla.\mathbf{u} = 0$ $\mathbf{K} = (\mu + \mu_T)(\nabla u + (\nabla u)^T)$ $\rho(\mathbf{u}.\nabla)k = \nabla\left[(\mu + \frac{\mu_T}{\sigma_k})\nabla k\right] + P_k - \rho\epsilon$ $\rho(\mathbf{u}.\nabla)\epsilon = \nabla\left[(\mu + \frac{\mu_T}{\sigma_\epsilon})\nabla\epsilon\right] + c_{\epsilon 1}\frac{\epsilon}{k}P_k - c_{\epsilon 2}\rho\frac{\epsilon^2}{k}, \epsilon = \epsilon p$ $\mu_T = \rho c_\mu \frac{k^2}{\epsilon}$ $P_k = \mu_T(\nabla u : (\nabla u + (\nabla u)^T)$
Shear Stress Equations	$\tau_{lam} = \mu\gamma \quad \gamma = \frac{dU}{dz}$ $\tau_{turb} = (\mu + \mu_T)\gamma \quad \gamma = \frac{dU}{dz}$

Boundary Condition	Values
Constant flow rate for μ-platform (m^3/s)	1.67[illegible], 7.33[illegible], 1.33[illegible]
Constant pressure (Pa)	0
No slip condition	
**Flow properties**	
Material	Water
Dynamic Viscosity (Pa.s)	0.0007
Density($kg.m^{-3}$)	993.37

Supp. Fig. 4 All system illustrations for 4 dynamic fluid systems, geometries and volume/surface area information used in simulations; **a** shaker, **b** spinner, **c** RCCS and **d** µ-platform. **e** Equations and variables used for CFD.

Supp. Table 1 Log2 transformation data of qRT-PCR analysis (** *p value < 0.05)*

	Day 30 +					Day 60 +					Day 120 +				
	Static	Spinner	Shaker	RCCS	µ-platform	Static	Spinner	Shaker	RCCS	µ-platform	Static	Spinner	Shaker	RCCS	µ-platform
CDH2	1.54**	2.71**	3.43**	0.82**	2.49**	3.42**	4.70**	2.34**	7.67**	2.84**	6.46**	7.16**	6.34**	6.54**	-2.00**
CTIP2	4.44**	4.14**	4.42**	6.92**	1.36**	-0.56**	2.06**	7.58**	3.65**	0.96**	0.03**	-0.43**	1.03**	4.97**	10.64**
FOXG1	-0.42**	2.65**	2.43**	-0.23**	2.27**	0.96**	-0.03	2.89**	2.24**	-1.60**	-2.12**	1.64**	0.72**	2.18**	4.73**
KROX20	1.02**	1.50**	2.30**	1.70**	2.05**	1.73**	3.57**	4.23**	5.64**	0.06**	2.05**	2.10**	0.82**	1.79**	5.42**
PAX6	-0.60**	1.43**	0.38**	-0.86**	2.30**	1.45**	2.70**	3.18**	0.14**	0.04**	0.88**	2.04**	1.57**	-0.25**	5.11**
PROX1	-0.10**	2.51**	1.25**	-2.94**	3.12**	0.43**	-1.15**	0.64**	-1.22**	-2.32**	-2.74**	6.71**	7.74**	7.86**	4.07**
PSD95	0.91**	1.63**	1.72**	0.25**	-0.60**	2.69**	-0.81**	1.60**	2.82**	0.82**	-0.22**	-0.03	-0.67**	0.94**	3.15**
SATB2	0.01	-0.09**	-0.81**	-1.60**	-1.47**	0.67**	0.07**	0.30**	1.75**	1.29**	-0.60**	-0.29**	3.58**	5.08**	5.49**
SOX1	-0.60**	0.14**	0.00	-1.00**	-1.43**	0.19**	-0.15**	0.15**	-0.43**	-2.56**	0.40**	2.46**	1.69**	0.59**	-0.94**
SOX2	0.48**	1.02**	1.06**	0.07**	0.67**	1.00**	1.80**	1.90**	1.49**	-5.06**	2.00**	4.48**	2.94**	1.29**	-0.64**
TBR1	0.79**	2.49**	1.79**	-5.06**	-6.64**	0.71**	-5.64**	-3.06**	-3.18**	-4.06**	-4.06**	-6.64**	-0.74**	0.94**	2.10**
TBR2	-2.25**	0.57**	-5.06**	-4.64**	-4.06**	-0.36**	-3.47**	-8.41**	-6.64**	-4.32**	-2.94**	-2.12**	-3.47**	-2.74**	1.51**
TTR	6.95**	7.10**	6.90**	8.99**	9.51**	6.94**	8.46**	7.89**	8.99**	-2.84**	8.34**	11.00**	8.86**	9.27**	0.06**
TUJ1	-0.36**	1.52**	1.04**	2.51**	2.74**	1.74**	1.69**	2.18**	4.12**	4.43**	-1.56**	-0.45**	-2.06**	-3.32**	-0.18**
CD11B	-1.43**	1.20**	3.20**	-1.36**	2.15**	-0.62**	1.08**	0.29**	1.63**	-5.64**	-5.06**	-5.06**	-4.64**	-1.64**	8.72**
GFAP	0.55**	1.51**	2.19**	1.14**	1.18**	2.40**	1.76**	0.59**	3.22**	-3.47**	-0.10**	0.26**	0.42**	0.05**	1.56**
IBA1	-0.60**	1.15**	0.65**	0.25**	-2.84**	1.76**	-0.47**	-2.06**	0.98**	3.72**	-2.40**	7.96**	3.80**	7.21**	4.00**
MAP2	0.56**	1.81**	1.90**	0.44**	0.90**	2.36**	4.50**	5.60**	4.75**	2.32**	5.68**	6.70**	6.17**	6.11**	1.93**
MBP	0.48**	0.64**	1.02**	1.55**	2.29**	0.02**	0.92**	2.10**	4.02**	-4.64**	-3.32**	-3.84**	-3.64**	1.29**	2.46**
NESTIN	-0.03**	0.21**	1.45**	1.94**	0.07**	1.20**	3.25**	6.77**	6.33**	-5.06**	2.39**	3.23**	1.71**	1.94**	2.92**
NEUN	4.06**	1.33**	0.52**	3.42**	-0.12**	0.67**	6.01**	6.19**	5.60**	4.32**	0.64**	1.29**	0.74**	2.51**	1.93**
OLIG2	0.16**	-1.15**	5.20**	-0.67**	3.16**	0.55**	0.01	1.53**	5.18**	-3.32**	2.37**	0.82**	3.75**	2.35**	5.09**
S100B	0.41**	1.49**	0.40**	-2.40**	-3.06**	2.31**	-1.15**	-2.25**	3.08**	-1.84**	4.25**	2.26**	1.03**	0.92**	4.53**
CD31	1.86**	1.32**	2.16**	-2.54**	-26.23**	-53.08**	-2.78**	-3.78**	-1.10**	-1.78**	2.87**	5.60**	11.69**	14.82**	46.53**
Ki-67	-1.65**	2.16**	-1.38**	2.25**	-1.95**	-3.75**	6.03**	8.44**	10.02**	11.64**	1.66**	6.25**	2.12**	11.10**	25.52**
b-catenin	1.14**	-2.35**	-1.27**	-1.42**	-1.16**	1.36**	2.51**	1.8**	2.59**	2.03**	-6.09**	10.72**	8.28**	13.12**	13.01**

Supp. Table 2 Antibody list for immunofluorescence and western blot analysis

Antibody	Host	Supplier/cat. no	IF dilution	WB dilution	FACS dilution	Region	Cellular localization
SOX2	Rabbit	Abcam, ab97959	1:200	1:1000	-	Radia glia/NSCs	Nucleus
PAX6	Rabbit	Abcam, ab195045	1:350	1:1000	-	Apical/Radia glia/NSCs	Nucleus
TUJ1	Mouse	Abcam, ab78078	1:1000	1:1000	-	Cortical neurons	Cytoplasm, cytoskeleton
N-CADHERIN	Mouse	Abcam, ab98952	1:500	1:1000	-	Apical epithelials	Cell membrane
FOXG1	Rabbit	Abcam, ab18259	1:200	1:1000	-	Forebrain	Nucleus
TBR1	Rabbit	Abcam, ab31940	1:200	1:1000	-	Preplate/Deep layer neurons	Nucleus
TBR2	Rabbit	Abcam, ab23345	1:500	1:1000	-	Intermediate progenitors	Nucleus

PROX1	Rabbit	Abcam, ab101851	1:750	1:1000	-	Hippocampus	Nucleus
CTIP2	Mouse	Abcam, ab233713	1:200	1:1000	-	Early born/Deep layer cortical neurons	Nucleus
SATB2	Rabbit	Abcam, ab34735	1:500	1:1000	-	Later born/Surface layer neurons	Nucleus matrix
PSD95	Mouse	Abcam, ab13552	1:200	1:1000	-	Postsynaptic marker	Cell membrane, junction, synapse
MAP2	Chk	Abcam, ab5392	1:1000	1:1000	-	Mature neurons	Cytoplasm, cytoskeleton
NEUN	Rabbit	Abcam, ab177487	1:200	1:1000	-	Neural marker	Nucleus, cytoplasm
CD45	Rabbit	Abcam, ab10558	-	-	1:1000	Mesodermal cell	Membrane
CD11B	Rat	Abcam, ab8878	1:500		1:1000	Mature microglia	Membrane
CD11B/C	Rabbit	Bioss, bs-1014R		1:1000	-	Mature microglia	Membrane
GFAP	Mouse	Abcam, ab10062	1:200	1:1000	1:1000	Mature astrocytes	Cytoplasm, intermediate filaments
MBP	Mouse	Abcam, ab62631	1:1500	1:2500	-	Mature oligodendrocytes	Myelin membrane. cytoplasmic side of myelin
NESTIN	Mouse	SantaCruz, sc-23927	1:500	1:1000	-	CNC progenitor cells	Intermediate filament
VGLUT1	Rabbit	Thermo, 48-2400	1:500	1:250	-	Glutamatergic neurons	Vesicular membranes
GABA-A	Rabbit	Affinity, Ab6207	1:250	1:500	-	GABAergic interneurons	Cytoplasm, cell membrane
Ki-67	Mouse	Cell signaling, 9449	1:500	1:500	-	Proliferating cells	Nucleus
B-catenin	Mouse	SantaCruz, sc-7963	1:250	1:500	-	Cell junction, adhesion	Cell membrane Cytoskeleton
CD31	Rabbit	SantaCruz, sc-376764	1:200	1:1000	-	Endothelial cells	Cell membrane
Beta-Actin	Mouse	Thermo, MA1140	1:1000	1:2500	-	A non-muscle cytoskeletal protein	Cytoskeleton
Alexa Fluor®647 conjugated Anti-Rabbit IgG H&L	Goat	Abcam, ab150079	1:1000	-	1:1000	-	-
Alexa Fluor®594 conjugated Anti-Chicken IgY H&L	Goat	Abcam, ab150176	1:1000	-	-	-	-

Alexa Fluor®488 conjugated Anti-Mouse IgG H&L	Goat	Abcam, ab150113	1:1000	-	1:1000	-	-
Alexa Fluor®555 conjugated Anti-Rat IgG H&L	Goat	Abcam, ab150158	1:1000	-	-	-	-
Alexa Fluor®488 conjugated Anti-Rat IgG H&L	Goat	Abcam, ab150157	-	-	1:1000	-	-
HRP conjugated Anti-Rabbit IgG(H+L)	Goat	ProteinTech, SA00001-2	-	1:1000	-	-	-
HRP conjugated Anti-Mouse IgG(H+L)	Goat	ProteinTech, SA00001-1	-	1:1000	-	-	-

Supp. Table 3 Primer list for qRT-PCR analysis

**Gene**	**5ˊforward 3ˊ**	**5ˊreverse 3ˊ**
ACTB	AGAGCTACGAGCTGCCTGAC	CGTGGATGCCACAGGACT
GAPDH	CGTAGCTCAGGCCTCAAGAC	GCTGCGGGCTCAATTTATAG
HPRT1	GACCAGTCAACAGGGGACAT	GTGTCAATTATATCTTCCACAATCAAG
KLF4	TTCACACTGTCTTCCCGATG	CAGTTGGGAACTTGACCATGA
C-MYC	AAGAGGCGAACACACAACG	AAAAGCTCCGTTTTAGCTCGT
OCT4	GCTTCGGATTTCGTCTTCTC	CTTAGCCAGGTCCGAGGAT
SOX2	TTGCTGCCTCTTTAAGACTAGGA	TAAGCCTGGGGCTCAAACT
TUJ1 (TUBB3)	GACCGGACGGTGAGTCAG	CACGAGGGAAGAAGTTTTGC
PAX6	GCAGGAGGAAGTGTTTTGCT	ATGTTGCTGGGTTGGTGTG
PROX1	GAGGGTGGGAAAGGGGTTTT	TCAAACGGCACTGAGCTTGT
FOXG1	GCTTAAACGAAAATGACCCAGT	CCTAGGGCCAATAGCTCACA
CDH2 (Ncad)	CTCCATGTGCCGGATAGC	CGATTTCACCAGAAGCCTCTAC
KROX20	TCCAAAACGGCTTTTCTGAC	CGGTCATCATTTGCTCCTC
SOX1	TCTTTTGGGTTGGTTTGTTAATTT	AATATAACTCCGCCGTCTGAAG
PSD95	GACGGCCTACTTTACTCACAGC	CTCCAGGATTGGAGTTGAGC
TBR1	TAGCAGCGCTAACATCAGCA	CCGAGCCCTAAGCCTTAAA
CTIP2	GCTTTCCACCTACCAGACCC	ATCACGGATGAGTGAGGGTG
TTR	CCGGTGAATCCAAGTGTCCT	AGATGCCAAGTGCCTTCCAG
SATB2	GTCTTCTCGGCTCTTGGTGT	GTGTCTTCTTCTGGTGCGGA
MAP2	ATGACCCCCTCATCCAAAG	CATGTGGCCAGACTCAACAC
TBR2 (EOMES)	GAGTCGGCAGGTGGGTAG	TCTTCCGAGGGGAAGGTAA
NES	ACCTGTGCCAGCCTTTCTTA	GCCAAGGTAGGGGTACGG
NEUN (RBFOX3)	CCCAACAGAAAGGGCTGAC	CTGGGCTTCCTTCGTCCT

IBA1	CTCCAGCTTGGAGGAAAAGC	TGGAGGGCAGATCCTCATCA
CD11B	AGGACTCTGCCCAGACCAC	GTCGGGGATACTTCGCTGT
GFAP	CTTGCTTCAGCGGTCAGG	TGCATGAGGCAGGTGGTA
S100B	TGTAGACCCTAACCCGGAGG	TGCATGGATGAGGAACGCAT
OLIG2	TCGCATCCAGATTTTCGGGT	TCCATGGCGATGTTGAGGTC
MBP	CCCTGCACCTGTCAGTCC	GGCAGCTTTCTCACATACCG
CD31 (PECAM1)	TGAGTGGTGGGCTCAGATTG	TGAGTCTAGGTCGGGGAGTG
Ki-67	CTGACCCTGATGAGAGTGAGGGA	ACTCTGTAGGGTCGAGCAGG
b-catenin	TGTTAAATTCTTGGCTATTACGACA	CCACCACTAGCCAGTATGATGA
