# Supplementary material for: Spatio-temporal dynamics enhance cellular diversity, neuronal function and further maturation of human cerebral organoids
Source: Commun Biol. 2023 Feb 14;6:173. doi: 10.1038/s42003-023-04547-1 (PMC9926461; doi:10.1038/s42003-023-04547-1)
Supplement: Supplementary file 3 — Description of Additional Supplementary Files [file 42003_2023_4547_MOESM3_ESM.pdf]

Description of Additional Supplementary Files

File name: Supplementary Data 1

Description: The numerical source data for a percentage of organoid harvestability results (Fig. 2b graph), b plots of organoid sizes (Fig. 2c,d,e,f,g graphs), c extracellular glutamate concentrations of organoids (Fig. 5d graph), d percentage of apoptotic zone (Fig. 6d graph)

File name: Supplementary Data 2

Description: Biological process (Gene Ontology) enrichment dataset of upregulated genes in all maturation systems and time intervals. a Upregulated genes, b Network statistics and c Enriched Biological process (GO) terms (uploaded separately as an excel file)

File name: Supplementary Data 3

Description: Tissue expression (TISSUE) enrichment dataset of upregulated genes in all maturation systems and time intervals. a Upregulated genes, b Network statistics, and c Enriched Tissue expression (TISSUE) terms (uploaded separately as an excel file)
