# Supplementary material for: Spatio-temporal dynamics enhance cellular diversity, neuronal function and further maturation of human cerebral organoids
Source: Commun Biol. 2023 Feb 14;6:173. doi: 10.1038/s42003-023-04547-1 (PMC9926461; doi:10.1038/s42003-023-04547-1)
Supplement: Supplementary file 7 — Reporting summary [file 42003_2023_4547_MOESM7_ESM.pdf]

nature portfolio

Corresponding author(s): *Double-blind peer review submissions: write DBPR and your manuscript number here instead of author names.*

Last updated by author(s): Jan 22, 2023

Reporting Summary

Nature Portfolio wishes to improve the reproducibility of the work that we publish. This form provides structure for consistency and transparency in reporting. For further information on Nature Portfolio policies, see our Editorial Policies and the Editorial Policy Checklist.

Statistics

For all statistical analyses, confirm that the following items are present in the figure legend, table legend, main text, or Methods section.

n/a	Confirmed	
☐	☒	The exact sample size (*n*) for each experimental group/condition, given as a discrete number and unit of measurement
☐	☒	A statement on whether measurements were taken from distinct samples or whether the same sample was measured repeatedly
☐	☒	The statistical test(s) used AND whether they are one- or two-sided<br>*Only common tests should be described solely by name; describe more complex techniques in the Methods section.*
☐	☒	A description of all covariates tested
☐	☒	A description of any assumptions or corrections, such as tests of normality and adjustment for multiple comparisons
☐	☒	A full description of the statistical parameters including central tendency (e.g. means) or other basic estimates (e.g. regression coefficient) AND variation (e.g. standard deviation) or associated estimates of uncertainty (e.g. confidence intervals)
☐	☒	For null hypothesis testing, the test statistic (e.g. *F*, *t*, *r*) with confidence intervals, effect sizes, degrees of freedom and *P* value noted<br>*Give P values as exact values whenever suitable.*
☒	☐	For Bayesian analysis, information on the choice of priors and Markov chain Monte Carlo settings
☐	☒	For hierarchical and complex designs, identification of the appropriate level for tests and full reporting of outcomes
☒	☐	Estimates of effect sizes (e.g. Cohen's *d*, Pearson's *r*), indicating how they were calculated

Our web collection on statistics for biologists contains articles on many of the points above.

Software and code

Policy information about availability of computer code

Data collection	Open source data is used. The normalized RNA expression levels of the specific neuronal/glial cell markers in different cell types (microglia, oligodendrocytes, astrocytes and neurons) downloaded from RNA single cell type data (Human protein atlas database; https://www.proteinatlas.org/about/download; rna_single_cell_type.tsv.zip).
Data analysis	Biological Process (Gene Ontology) and Tissue expression (TISSUES) enrichments of upregulated genes (log2 transformation of fold regulation ≥ 1.9, p value < 0.05) of each system and day groups were realized via STRING v11.5 database (https://string-db.org/

For manuscripts utilizing custom algorithms or software that are central to the research but not yet described in published literature, software must be made available to editors and reviewers. We strongly encourage code deposition in a community repository (e.g. GitHub). See the Nature Portfolio guidelines for submitting code & software for further information.

Data

Policy information about availability of data

All manuscripts must include a data availability statement. This statement should provide the following information, where applicable:

- Accession codes, unique identifiers, or web links for publicly available datasets
- A description of any restrictions on data availability
- For clinical datasets or third party data, please ensure that the statement adheres to our policy

Source data for the presented figures are provided as Supp. Data 1-2-3 with this paper. Further simulation data that is generated and analyzed during the current

study is available from the corresponding author on reasonable request. Besides that, the normalized RNA expression levels of the specific neuronal/glial cell markers in different cell types downloaded from RNA single cell type data from Human protein atlas database; https://www.proteinatlas.org/about/download; rna_single_cell_type.tsv.zip. Also, Biological Process (Gene Ontology) and Tissue expression (TISSUES) enrichments of upregulated genes of each system and day groups were realized via STRING v11.5 database (https://string-db.org/).

Human research participants

Policy information about studies involving human research participants and Sex and Gender in Research.

Reporting on sex and gender	Human research participants were not involved
Population characteristics	*Describe the covariate-relevant population characteristics of the human research participants (e.g. age, genotypic information, past and current diagnosis and treatment categories). If you filled out the behavioural & social sciences study design questions and have nothing to add here, write "See above."*
Recruitment	*Describe how participants were recruited. Outline any potential self-selection bias or other biases that may be present and how these are likely to impact results.*
Ethics oversight	*Identify the organization(s) that approved the study protocol.*

Note that full information on the approval of the study protocol must also be provided in the manuscript.

Field-specific reporting

Please select the one below that is the best fit for your research. If you are not sure, read the appropriate sections before making your selection.

☒ Life sciences ☐ Behavioural & social sciences ☐ Ecological, evolutionary & environmental sciences

For a reference copy of the document with all sections, see nature.com/documents/nr-reporting-summary-flat.pdf

Life sciences study design

All studies must disclose on these points even when the disclosure is negative.

Sample size	Overall, 2 replicates for each of the maturation processes, whereas each replicate contained 40 embryoid bodies , which were matured as organoids. With respect to plots of organoid sizes, n=8-40 and for other characterization analyses, n=3
Data exclusions	For Biological Process (Gene Ontology) and Tissue expression (TISSUES) enrichments of upregulated genes (log2 transformation of fold regulation ≥ 1.9, p value < 0.05) of each system
Replication	Independent replicates of 2 for each of the maturation processes, whereas 4 for µ-platform system.
Randomization	Matured organoids harvested from each of the processes were randomly analyzed for characterization
Blinding	Samples were blinded subsequent to all characterization tests such as size analysis, qRT-PCR, western blot, histological analysis

Reporting for specific materials, systems and methods

We require information from authors about some types of materials, experimental systems and methods used in many studies. Here, indicate whether each material, system or method listed is relevant to your study. If you are not sure if a list item applies to your research, read the appropriate section before selecting a response.

Materials & experimental systems

n/a	Involved in the study	
☐	☒	Antibodies
☐	☒	Eukaryotic cell lines
☒	☐	Palaeontology and archaeology
☒	☐	Animals and other organisms
☒	☐	Clinical data
☒	☐	Dual use research of concern

Methods

n/a	Involved in the study	
☒	☐	ChIP-seq
☒	☐	Flow cytometry
☒	☐	MRI-based neuroimaging

Antibodies

Antibodies used

Antibody list for immunofluorescence and western blot analysis
Antibody, Host Supplier/cat. no, IF dilution, WB dilution, Region Cellular localization
SOX2 Rabbit Abcam, ab97959 1:200 1:1000 Radia glia/NSCs Nucleus
PAX6 Rabbit Abcam, ab195045 1:350 1:1000 Apical/Radia glia/NSCs Nucleus
TUJ1 Mouse Abcam, ab78078 1:1000 1:1000 Cortical neurons Cytoplasm, cytoskeleton
N-CADHERIN Mouse Abcam, ab98952 1:500 1:1000 Apical epithelials Cell membrane
FOXG1 Rabbit Abcam, ab18259 1:200 1:1000 Forebrain Nucleus
TBR1 Rabbit Abcam, ab31940 1:200 1:1000 Preplate/Deep layer neurons Nucleus
TBR2 Rabbit Abcam, ab23345 1:500 1:1000 Intermediate progenitors Nucleus
PROX1 Rabbit Abcam, ab101851 1:750 1:1000 Hippocampus Nucleus
CTIP2 Mouse Abcam, ab233713 1:200 1:1000 Early born/Deep layer cortical neurons Nucleus
SATB2 Rabbit Abcam, ab34735 1:500 1:1000 Later born/Surface layer neurons Nucleus matrix
PSD95 Mouse Abcam, ab13552 1:200 1:1000 Postsynaptic marker Cell membrane, junction, synapse
MAP2 Chk Abcam, ab5392 1:1000 1:1000 Mature neurons Cytoplasm, cytoskeleton
NEUN Rabbit Abcam, ab177487 1:200 1:1000 Neural marker Nucleus, cytoplasm
CD11B Rat Abcam, ab8878 1:500 Mature microglias Membrane
CD11B/C Rabbit Bioss, bs-1014R 1:1000 Mature microglias Membrane
GFAP Mouse Abcam, ab10062 1:200 1:1000 Mature astrocytes Cytoplasm, intermediate filaments
MBP Mouse Abcam, ab62631 1:1500 1:2500 Mature oligodendrocytes Myelin membrane. cytoplasmic side of myelin
NESTIN Mouse SantaCruz, sc-23927 1:500 1:1000 CNC progenitor cells Intermediate filament
VGLUT1 Rabbit Thermo, 48-2400 1:500 1:250 Glutamatergic cells Vesicular membranes
Ki67 Mouse SantaCruz, sc-23900 1:200 1:1000 Proliferating cells Nucleus
CD31 Rabbit SantaCruz, sc-376764 1:200 1:1000 Endothelial cells Cell membrane
Beta-Actin Mouse Thermo, MA1140 1:1000 1:2500 A non-muscle cytoskeletal protein Cytoskeleton
Alexa Fluor®647 conjugated Anti-Rabbit IgG H&L Goat Abcam, ab150079 1:1000 - - -
Alexa Fluor®594 conjugated Anti- Chicken IgY H&L Goat Abcam, ab150176 1:1000 - - -
Alexa Fluor®488 conjugated Anti- Mouse IgG H&L Goat Abcam, ab150113 1:1000 - - -
Alexa Fluor®555 conjugated Anti-Rat IgG H&L Goat Abcam, ab150158 1:1000 - - -
HRP conjugated Anti-Rabbit IgG(H+L) Goat ProteinTech, SA00001-2 - 1:1000 - -
HRP conjugated Anti-Mouse IgG(H+L) Goat ProteinTech, SA00001-1 - 1:1000 - -

Validation

We applied validation statements on the manufacturer's website for the listed antibodies above

Eukaryotic cell lines

Policy information about cell lines and Sex and Gender in Research

Cell line source(s)

iPSC lines that were previously reprogrammed from human dermal fibroblasts of healthy donors and characterized in terms of pluripotency markers and mycoplasma purity, were obtained from Izmir Biomedicine and Genome Center, Stem Cell and Organoid Technologies Laboratory

Authentication

iPSCs was phenotypically authenticated with Sanger DNA sequencing analysis

Mycoplasma contamination

Mycoplasma purity was confirmed by Stem Cell and Organoid Technologies Laboratory, Izmir Biomedicine and Genome Center,

Commonly misidentified lines
(See ICLAC register)

There are no misidentified cell lines used in this study
